# Herpes zoster correlates with pyogenic liver abscesses in Taiwan

**DOI:** 10.7603/s40681-016-0022-4

**Published:** 2016-11-17

**Authors:** Shen Mei-Ling, Liao Kuan-Fu, Tsai Sung-Mao, Lin MS Cheng-Li, Lai Shih-Wei

**Affiliations:** 1 Department of Anesthesiology, Taichung Tzu Chi General Hospital, Taichung, 427, Taiwan; 2 College of Medicine, Tzu Chi University, Hualien, 970, Taiwan; 3 Department of Internal Medicine, Taichung Tzu Chi General Hospital, Taichung, 427, Taiwan; 4 Graduate Institute of Integrated Medicine, China Medical University, Taichung, 404, Taiwan; 5 College of Medicine, China Medical University, Taichung, 404, Taiwan; 6 Management Office for Health Data, China Medical University Hospital, Taichung, 404, Taiwan; 7 Department of Family Medicine, China Medical University Hospital, No. 2, Yuh-Der Road, Taichung, 404, Taiwan

**Keywords:** Herpes zoster, Pyogenic liver abscess, Biliary stone, Chronic kidney disease, Chronic liver disease, Cancers, Diabetes mellitus

## Abstract

**Objective:**

The purpose of the paper was to explore the relationship between herpes zoster and pyogenic liver abscesses in Taiwan.

**Methods:**

This was a nationwide cohort study. Using the database of the Taiwan National Health Insurance Program, there were 33049 subjects aged 20-84 years who were newly diagnosed with herpes zoster from 1998 to 2010 that were selected for our study, and they were our herpes zoster group. 131707 randomly selected subjects without herpes zoster were our non-herpes zoster group. Both groups were matched by sex, age, other comorbidities, and the index year of their herpes zoster diagnosis. The incidence of pyogenic liver abscesses at the end of 2011 was then estimated. The multivariable Cox proportional hazard regression model was used to estimate the hazard ratio and 95% confidence interval for pyogenic liver abscesses associated with herpes zoster and other comorbidities.

**Results:**

The overall incidence rate was 1.38-fold higher in the herpes zoster group than in the non-herpes zoster group (4.47 *vs*. 3.25 per 10000 person-years, 95% confidence interval 1.32, 1.44). After controlling for potential confounding factors, the adjusted hazard ratio of pyogenic liver abscesses was 1.34 in the herpes zoster group (95% confidence interval 1.05, 1.72) when compared with the non-herpes zoster group. Sex (in this case male), age, presence of biliary stones, chronic kidney diseases, chronic liver diseases, cancers, and diabetes mellitus were also significantly associated with pyogenic liver abscesses.

**Conclusions:**

Patients with herpes zoster are associated with an increased hazard of developing pyogenic liver abscesses.

## 1. Introduction

Although the incidence of pyogenic liver abscesses was considered rare in the past, the condition, related to underlying hepatobiliary diseases and polymicrobial infection, often leads to severe illness and death.[1] Recently, primary liver abscesses (or cryptogenic liver abscesses), without any underlying hepatobiliary diseases, have been considered as pyogenic liver abscesses. [2, 3] However, Klebsiella pneumoniae has been indicated to be the first most common pathogen to the development of pyogenic liver abscesses, with Escherichia coli as the second most common pathogen. Diabetes, biliary disease, renal diseases, and cancers have been found to be the high predisposing comorbidities for pyogenic liver abscess. Chronic kidney disease and diabetes are definitely known to result in a compromised immune system and are considered to be risk factors for some infectious diseases.[4, 5]

The cause of herpes zoster is known to be the reactivation of the varicella-zoster virus, which occurs in cases of declined cellmediated immunity. Less than 5% of the population will be attacked by a second episode of herpes zoster, and second episodes that do occur frequently do so more in immune-compromised populations, like the aged, those with chronic renal disease, diabetes, malignancies, organ transplantation, or HIV infection.[6, 7] So we supposed that herpes zoster might be related to pyogenic liver abscessed. In the study, then, we used data gathered by the Taiwan National Health Insurance (NHI) program to clarify the relationship between herpes zoster and pyogenic liver abscess.

## 2. Methods

### 2.1. Study design and population

We conducted a nationwide cohort study using the database of the Taiwan National Health Insurance Program. The program began in March 1995 and covers almost 99% of the 23 million residents living in Taiwan.[8] The details of the program can be found in previous high-quality studies.[9-13] The study was approved by the Institutional Review Board (IRB) of China Medical University and Hospital in Taiwan (CMUH-104-REC2-115).

### 2.2. Study participants

We identified subjects aged 20-84 years with newly-diagnosed herpes zoster during the period of 1998-2010 as the herpes zoster group, based on the International Classification of Diseases 9th Revision (ICD-9 code 053). The date of herpes zoster diagnosis was defined as the index date. For each subject with herpes zoster, four comparison subjects without herpes zoster were randomly selected from the same NHI database as the non-herpes zoster group. The non-herpes zoster subjects were matched with the herpes zoster subjects by sex, age (every 5-year span), comorbidities, and the index year of the herpes zoster diagnosis. Subjects with history of pyogenic liver abscesses (ICD-9 code 572.0), amebic liver abscesses (ICD-9 code 006.3), or liver transplantation (ICD-9 codes 996.82 and V42.7) before the index date were excluded from this study.

### 2.3. Potential comorbidities and main outcome measurement

Comorbidities potentially associated with pyogenic liver abscesses were selected in the study as follows: alcohol-related diseases (ICD-9 codes 291, 303, 305.00, 305.01, 305.02, 305.03, 571.0- 571.3, 790.3 and V11.3), biliary stones (ICD-9 code 574), chronic kidney diseases (ICD-9 codes 585–586 and 588.8–588.9), cancers (ICD-9 codes 140-208), diabetes mellitus (ICD-9 code 250), as well as chronic liver diseases (including cirrhosis (ICD-9 codes 571.5 and 571.6), Hepatitis B (ICD-9 codes V02.61, 070.20, 070.22, 070.30 and 070.32), Hepatitis C (ICD-9 codes V02.62, 070.41, 070.44, 070.51 and 070.54) and other chronic hepatitis (ICD-9 codes 571.40, 571.41, 571.49, 571.8 and 571.9).

The main outcome was a new primary diagnosis of pyogenic liver abscesses based on hospital discharge registries during the follow-up periods of subjects. Each subject was monitored from their index date until they were diagnosed with pyogenic liver abscesses, or else they had to be censored because of a failure of the subject to follow-up, death, their withdrawal from insurance, or the end of the period of study (December 31, 2011).

### 2.4. Statistical analysis

The distributions of sex, age, and other comorbidities were compared between the herpes zoster group and the non-herpes zoster group using a *Chi*-square test for categorized variables and a t-test for continuous variables. The incidence of pyogenic liver abscesses was estimated as the number of pyogenic liver abscesses events identified during the follow-up period, divided by the total follow-up person-years for each group. Initially, we included all variables in our univariable Cox proportional hazards regression model. Those found to be significant in the univariable model were further included in our multivariable Cox proportional hazards regression model to estimate the hazard ratio (HR) and 95 % confidence interval (CI) of the pyogenic liver abscesses risk associated with herpes zoster and other comorbidities. The statistical significance level was set to a two-sided probability value & 0.05. All analyses were performed using SAS software version 9.2 (SAS Institute Inc., Cary, North Carolina, USA).

## 3. Results

### 3.1. Baseline characteristics of the study population

Table 1 reveals the baseline characteristics between the herpes zoster group and the non-herpes zoster group. There were 33049 subjects in the herpes zoster group and 131707 subjects in the non-herpes zoster group, with similar distributions of sex and age. The mean ages (standard deviation) of the study subjects were 54.8 ±16.2 years for the herpes zoster group and 54.3 ± 16.3 for the non-herpes zoster group. The mean follow-up periods (standard deviation) were 5.68 ± 3.33 years in the herpes zoster group and 5.64 ± 3.32 years in the non-herpes zoster group. No significant difference was noted regarding the distribution of other comorbidities between the herpes zoster group and the non-herpes zoster group (*P* > 0.05 for all).

### 3.2. Incidence of pyogenic liver abscesses in the study population

The follow-up results revealed that the overall incidence of pyogenic liver abscesses was 1.38-fold greater in the herpes zoster group than in the non-herpes zoster group (4.47 *vs.* 3.25 per 10000 person-years, 95% CI 1.32, 1.44). Because no subject developed pyogenic liver abscesses among the herpes zoster group that was aged 20-39 years, subjects aged 20-39 years and aged 40- 64 years were merged into a single cohort of subjects aged 20-64 years. The incidence rates of pyogenic liver abscesses, as stratified by sex, age and follow-up period, were all higher in the herpes zoster group than those in the non-herpes zoster group. The herpes zoster group aged 65-84 years had the highest incidence rate of pyogenic liver abscesses (8.38 per 10000 person-years). The analysis as stratified by the follow-up periods revealed that the risk of pyogenic liver abscesses persisted over time, even one year after herpes zoster diagnosis (Table 2).

### 3.3. Pyogenic liver abscesses associated with herpes zoster and other comorbidities

After controlling for potential confounding factors, our multivariable Cox proportional hazards regression model revealed that the adjusted HR of pyogenic liver abscesses was 1.34 (95% CI 1.05, 1.72) for the herpes zoster group, when compared with the nonherpes zoster group. Sex (in this case male) (adjusted HR 1.41, 95% CI 1.13, 1.75), age (per one year, adjusted HR 1.03, 95% CI 1.02, 1.04), biliary stones (adjusted HR 2.87, 95% CI 2.03, 4.06), chronic kidney diseases (adjusted HR 2.41, 95% CI 1.60, 3.63), chronic liver diseases (adjusted HR 1.31, 95% CI 1.01, 1.71), cancers (adjusted HR 1.60, 95% CI 1.02, 2.53), and diabetes mellitus (adjusted HR 1.66, 95% CI 1.30, 2.12) were also significantly associated with pyogenic liver abscesses (Table 3).

**Table 1:** 

	Non-herpes zoster N = 131707		Herpes zoster N = 33049		
Variable	n	(%)	n	(%)	*P* value*
Sex					0.94
Female	69710	52.9	17500	53.0	
Male	61997	47.1	15549	47.1	
Age group (year)					0.92
20-39	26484	20.1	6625	20.1	
40-64	65560	49.8	16434	49.7	
65-84	39663	30.1	9990	30.2	
Age (year), mean (standard deviation)†	54.3	16.3	54.8	16.2	0.001
Follow-up time (year), mean (standard deviation)†	5.64	3.32	5.68	3.33	0.02
Baseline comorbidities					
Alcohol-related diseases	4184	3.18	1111	3.36	0.09
Biliary stones	4572	3.47	1210	3.66	0.09
Chronic kidney diseases	3699	2.81	986	2.98	0.09
Chronic liver diseases	23343	17.7	5906	17.9	0.53
Cancers	5818	4.42	1521	4.60	0.15
Diabetes mellitus	23589	17.9	5967	18.1	0.54

Data are presented as the number of subjects in each group with percentages given to their right, or the mean with the standard deviation given to the right.

*Chi-square test and †*t-test* comparing subjects with and without herpes zoster.

**Table 2:** 

	Non-herpes zoster				Herpes zoster				
			Person-				Person-		
Variable	N	Event			N	Event			IRR* (95% CI)
			years	Rate†			years	Rate†	
All	131707	241	742588	3.25	33049	84	187870	4.47	1.38 (1.32, 1.44)
Sex									
Female	69710	109	398992	2.73	17500	39	100678	3.87	1.42 (1.34, 1.50)
Male	61997	132	343596	3.84	15549	45	87192	5.16	1.34 (1.27, 1.43)
Age group (year)									
20-64**	992044	134	537146	2.49	23059	40	135360	2.96	1.18 (1.12, 1.25)
65-84	39663	107	205442	5.21	9990	44	52510	8.38	1.61 (1.50, 1.73)
Follow-up period									
≤ 6 months	131707	20	65489	3.05	33049	5	16438	3.04	1.00 (0.94, 1.05)
6-12 months	130187	21	64563	3.25	32686	7	16224	4.31	1.33 (1.26, 1.39)
> 1 year	127529	200	612536	3.27	32063	72	155208	4.64	1.42 (1.36, 1.48)

*IRR (incidence rate ratio): herpes zoster vs. non-herpes zoster (95% CI)

†Incidence rate: per 10000 person-years.

** Because no subject developed pyogenic liver abscesses among the herpes zoster group aged 20-39 years, subjects aged 20-39 years and aged 40-64 years were merged into subjects aged 20-64 years.

**Table 3:** 

	Crude		Adjusted†	
Variable	HR	(95%CI)	HR	(95%CI)
Sex (male *vs.* female)	1.39	(1.12, 1.73)	1.41	(1.13, 1.75)
Age (per one year)	1.04	(1.03, 1.05)	1.03	(1.02, 1.04)
Baseline comorbidities (yes vs. no)				
Herpes zoster	1.38	(1.07, 1.77)	1.34	(1.05, 1.72)
Alcohol-related diseases	1.69	(0.95, 3.00)	-	-
Biliary stones	4.38	(3.13, 6.12)	2.87	(2.03, 4.06)
Chronic kidney diseases	4.08	(2.73, 6.09)	2.41	(1.60, 3.63)
Chronic liver diseases	1.82	(1.41, 2.34)	1.31	(1.01, 1.71)
Cancers	2.04	(1.30, 3.21)	1.60	(1.02, 2.53)
Diabetes mellitus	2.57	(2.04, 3.24)	1.66	(1.30, 2.12)

†Controlling for sex, age, biliary stones, chronic kidney diseases, chronic liver diseases, cancers, and diabetes mellitus.

## 4. Discussion

Pyogenic liver abscesses are defined as a common health problem in Taiwan society, with the incidence rate being higher than those in western society. According to the results of select studies, the annual incidence rate of pyogenic liver abscesses in Canada, United States and Denmark was only 2.3 × 10^-6^, 3.6 × 10^-6^ and 1.1 × 10^-6^ in 2003, 2005, and 2006 respectively, but in Taiwan it was 17.5 × 10^-6^ in 2004.[14-16] However, large-scale populationbased studies, which have been conducted to explore the relationship between herpes zoster and subsequent pyogenic liver abscesses, have thus far been few. In our study, we found that the adjusted HR of pyogenic liver abscesses was 1.34 (95% CI 1.05, 1.72), which is similar to other risk factors. Sex (in this case male) (adjusted HR 1.41, 95% CI 1.13, 1.75), age (per one year, adjusted HR 1.03, 95% CI 1.02, 1.04), biliary stones (adjusted HR 2.87, 95% CI 2.03, 4.06), chronic kidney diseases (adjusted HR 2.41, 95% CI 1.60, 3.63), chronic liver diseases (adjusted HR 1.31, 95% CI 1.01, 1.71), cancers (adjusted HR 1.60, 95% CI 1.02, 2.53), and diabetes mellitus (adjusted HR 1.66, 95% CI 1.30, 2.12) were also significantly associated with pyogenic liver abscesses.

Before the 1980s, the predominant cause of pyogenic liver abscesses was Klebsiella pneumoniae infection in Asian countries and areas[17-21]. According to our knowledge, Klebsiella pneumoniae infections are commonly associated to diabetes mellitus. Diabetes mellitus, which can decrease chemotaxis and phagocytosis and increase the susceptibility of bacterial infections to affect neutrophil functions, has been signaled as the strongest risk factor for liver abscesses in Taiwan.[22, 23] The condition of neutrophil dysfunction can be found in chronic kidney disease patients, especially the end stage of renal disease with dialysis population, which have a higher risk of infection.[15, 24] Several studies have reported that underlying chronic biliary diseases were the major cause of pyogenic liver abscesses.[15, 24] The bacteria might remain in the biliary tract and exacerbate the infection in patients with chronic biliary diseases. Kang and Hwang found that the incidence rate of gastroenterological cancers was higher in aged patients (>65 years) as was the incidence of pyogenic liver abscesses.[25] Hepatocellular carcinoma is associated to pyogenic liver abscesses in patients, especially in older patients. 65 years or older has been found to be one of the independent risk factors for pyogenic liver abscesses.[25-27]

Herpes zoster results from the reactivation of the latent varicella- zoster virus. The incidence of herpes zoster usually increases with age and other immune-compromised conditions, such as diabetes mellitus, chronic kidney diseases, and so on. Another factor is that cellular immunity is also decreased in advanced age individuals.[28] Diabetes mellitus and chronic kidney diseases may result in a compromised immune system and neutrophil dysfunction, so these conditions may be risk factors of infectious diseases.[29-31]

In our study, we supposed that the mechanisms underlying the association between herpes and subsequent pyogenic liver abscesses may be complicated. First, the varicella-zoster virus possibly affects various liver tissues *via* neural, hematologic, or extrinsic spread. Second, it is possible that the reactivation of the varicella-zoster virus triggers some immunologic mechanisms, such as compromising cellular immunity and creating neutrophil dysfunction, which may later lead to pyogenic liver abscesses. Third, the initiation of emergence of herpes zoster and pyogenic liver abscesses could be the result of any dysfunction of the host’s immunity, which is tasked with keeping the latent varicella-zoster virus under control and maintaining the healthy condition of the liver. Fourth, both herpes zoster and pyogenic liver abscesses could be associated with similar comorbidities or risk factors such as increased age and disorders which decrease the immune competency of an individual. The latter includes conditions such as diabetes mellitus, chronic renal diseases, chronic liver diseases, malignancies, *etc*. Hence, patients who have these underlying conditions will therefore be expected to develop herpes zoster or pyogenic liver abscesses. This could partially explain the biological plausibility of the observed association between herpes zoster and pyogenic liver abscess seen in this study.

Having said all of that, there are some limitations to our study. First, several studies have obtained their diagnoses through a standardized clinical procedure but our study obtained diagnoses of herpes zoster and pyogenic liver abscesses dependent upon ICD-9 codes. The diagnosis accuracy of herpes zoster pyogenic liver abscess and other comorbidities studied based on the IDC 9 codes has been thoroughly discussed in previous studies.[32-37] Second, some patients with herpes zoster may not go for medical help. This small percentage of individuals with herpes zoster who do not go for medical help would be erroneously categorized as non-herpes zoster. Third, due to the natural limitation of the national insurance database used in our study, there was no record of the severity of herpes zoster and pyogenic liver abscesses, nor the duration of other risk factors. In addition, there is no information available about the treatment of these risk factors and how that would impact the risk of either herpes zoster or pyogenic liver abscesses.

Nevertheless, we have concluded that patients with herpes zoster are associated with an increased hazard of developing pyogenic liver abscesses. The findings in our study suggest that a patient’s hepatic condition should be cared for after they are diagnosed with herpes zoster.
